# Impact of Heart Failure on Recovery After Traumatic Brain Injury in Adults 65 and Older

**DOI:** 10.1093/geroni/igaf122.3960

**Published:** 2025-12-31

**Authors:** Sofio Dumbadze, Jennifer Albrecht, Jason Falvey

**Affiliations:** University of Maryland, Baltimore, Baltimore, Maryland, United States; University of Maryland, Baltimore, Maryland, United States; University of Maryland School of Medicine, Baltimore, Maryland, United States

## Abstract

Heart failure (HF) is common among older adults with traumatic brain injury (TBI). Understanding how HF affects days spent at home, a meaningful measure of recovery following an acute event, can inform care management and improve outcomes in this vulnerable population. We examined the impact of HF on days at home following TBI among adults aged 65 and older, hypothesizing that people with HF would spend fewer days at home after TBI compared to those without HF. We conducted a retrospective cohort study among adults aged 65 and older who were hospitalized with TBI using Medicare administrative claims data from 2010 to 2018. TBI and HF were identified using disease classification codes. We measured days at home over 1-year post-TBI by subtracting the number of days spent in inpatient care, skilled nursing facilities, nursing homes, emergency or outpatient observation settings, and the number of days spent deceased from 365. We modeled days at home as a function of HF using a negative binomial model. Among 20,206 older adults with TBI, 9,056 (44.8%) had HF. After TBI, older adults with HF spent fewer days at home than without HF (adjusted rate ratio 0.96; 95% confidence interval 0.94-0.99). This translates to an adjusted difference of 9 fewer days at home over the year following TBI among older adults with HF. Integration of chronic disease management into rehabilitation pathways for TBI survivors with HF may be warranted.

